# Tip110 Expression Facilitates the Release of HEXIM1 and pTEFb from the 7SK Ribonucleoprotein Complex Involving Regulation of the Intracellular Redox Level

**DOI:** 10.14336/AD.2021.0528

**Published:** 2021-12-01

**Authors:** Ying Liu, Lu Li, Khalid Timani, Carl White, Johnny J He

**Affiliations:** ^1^Department of Microbiology and Immunology,; ^2^Center for Cancer Cell Biology, Immunology and Infection, and; ^3^Department of Physiology and Biophysics, Rosalind Franklin University of Medicine and Science Chicago Medical School, 3333 Green Bay Road, North Chicago, IL 60064, USA.

**Keywords:** Tip110, HEXM1, pTEFb, 7SK, CDK9, Nuclear matrix

## Abstract

HIV-1 Tat-interacting protein of 110 kDa (Tip110; p110^nrb^/SART3) has been identified to be important for HIV gene transcription and several host gene expression. In this study, we showed that Tip110 was present in the 7SK snRNP through direct binding to MEPCE, a component of the 7SK snRNP complex. In addition, we found a positive association between Tip110 expression, change of HEXIM1 from dimer/oligomer to monomer, and release of HEXIM1 and P-TEFb from the 7SK snRNP complex. A similar association was also noted specifically in nuclear matrix as well as in chromatin where the free HEXIM1 and 7SK snRNP-bound HEXIM1 are located. Moreover, we demonstrated that Tip110 expression was linked to the glutathione metabolic pathway and the intracellular redox level, which in turn regulated HEXIM1 dimerization/oligomerization. Lastly, we performed the FRET microscopic analysis and confirmed the direct relationship between Tip110 expression and HEXIM1 dimerization/oligomerization *in vivo*. Taken together, these results identified a new mechanism governing HEXIM1 dimerization/oligomerization and the release of HEXIM1 and P-TEFb from the 7SK snRNP complex. These results also yield new insights to the roles of Tip110 in HIV gene transcription and replication.

Positive transcription elongation factor b (P-TEFb) is composed of cyclin T and cyclin-dependent kinase 9 (CDK9). It phosphorylates serine residues in the heptad repeats Tyr-Ser-Pro-Thr-Ser-Pro-Ser of the carboxyl-terminal domain (CTD) of the largest subunit of RNA polymerase II (RNAPII), which results in the transition from abortive to productive elongation and subsequent transcription of HIV full-length transcripts [1-4]. There are two forms of P-TEFb in cells. One is active P-TEFb. It binds to other cellular proteins including transcriptional factors and regulates host gene expression [5-13]. This form P-TEFb also binds to viral proteins such as EBV E2, HSV VP16, HTLV Tax and HIV Tat [14-17]. The other form is a larger, inactive form P-TEFb located in the small nuclear 7SK ribonucleoprotein complex (7SK snRNP) [18, 19]. The 7SK snRNP complex consists of hexamethylene bis-acetamide inducible protein 1 (HEXIM1), methylphosphate capping enzyme (MEPCE), La ribonucleoprotein 7 (LARP7), and a 7SK small nuclear RNA (snRNA), each of the components has unique roles in the complex formation and contributes to the complex function [4, 20, 21]. Binding of cellular and viral proteins to inactive P-TEFb in the 7SK RNP complex leads to the release of P-TEFb from the 7SK snRNP complex to become active [4, 21]. Post-translational modifications of P-TEFb or components of the 7SK snRNP are also possibilities [10, 16, 22-25]. In actively proliferating cells, about 50-80% of P-TEFb is in the inactive 7SK snRNP form [12-14, 26]. However, the mechanisms about the release of P-TEFb from the 7SK snRNP are not fully understood.

HEXIM1 has been shown to play important roles in regulating the dynamic balance between the active form P-TEFb and the 7SK RNP complex-bound inactive p-TEFb. HEXIM1 is a nuclear protein, it is rapidly induced in human cells treated with hexamethylene bisacetamid [27], a potent inducer of cell differentiation and suppressor of cell proliferation [28]. The primary sequence of HEXIM1 can be grouped into four distinct regions. They include N terminus (aa 1-149), arginine-rich basic region (150-163) that contains the nuclear localization signal and binds the 7SK snRNA, negatively charged central region (aa 164-254), and C terminus (aa 255-359) that contains a coiled-coil leucine zipper for dimerization and binds to cyclin T [24, 29, 30-32]. HEXIM1 binds 7SK snRNA and cyclin T of P-TEFb, facilitates the assembly of the 7SK snRNP complex, and inactivates P-TEFb in the 7SK snRNP complex [19, 31]. Thus, HEXIM1 is also called a P-TEFb inhibitor. HEXIM1 exists as dimers or oligomers *in vivo*, either alone or in the 7SK snRNP complex [30]. These dimers/oligomers are very stable under high salt conditions and cannot be disassembled by stress-inducing agents or by degradation of the 7SK RNA scaffold [30]. The release of HEXIM1 and P-TEFb has been associated with a conformational change of 7SK snRNA and binding of several heterogeneous ribonucleoproteins (hnRNP) such as hnRNPA1, A2, Q and R [23]. But, there is no definitive evidence to support that the release of HEXIM1 precedes the release of T-TEFb from the 7SK snRNP complex or the release of both HEXIM1 and p-TEFb from the 7SK snRNP complex occurs simultaneously.

In an early study, we show that Tat-interacting protein of 110 KDa (Tip110) directly interacts with HIV Tat protein and enhances Tat-mediated HIV transcription and replication [33]. Subsequently, we show that Tip110 binds to the unphosphorylated RNAPII CTD and increases P-TEFb recruitment to the Tat.RNAPII complex and phosphorylation of the RNAPII CTD [34]. In the current study, we used a combination of molecular, cellular and biochemical approaches and uncovered a potential new regulatory role of Tip110 in the release of P-TEFb and HEXM1 from the 7SK snRNP complex.

## MATERIALS AND METHODS

### Cell Lines and Cell Transfections

293T and Hela were purchased from the American Tissue Culture Collection and were cultured in Dulbecco's modified Eagle's medium with 10% fetal bovine serum, at 37 °C with 5% CO_2_. Transfections were carried out using the standard calcium phosphate precipitation method.

### Plasmid constructions

Venus.HEXIM1 and Cerulean.HEXIM1 constructs were created by cloning of the wild-type HEXIM1 into mVenus.N1 and mCerulean.N1 expression vectors (Addgene). Briefly, the Hexim1 insert was obtained through EcoRI and EcoRV digestion on pCMV.Hexim1 (OriGene Technololigies Inc.) and then ligated to mVenus.N1 and mCerulene.N1 vectors that were digested by EcoRI and EcoRV. Cerulean-5-Venus expressing a Cerulean/Venus fusion protein was purchased from Addgene. Tip110.His, psh-Ctrl, and psh-Tip110 plasmids were described elsewhere [23, 34]. pMEPCE.Flag and pGST.MEPCE plasmids were kindly provided by Dr. Qiang Zhou of University of California, Berkeley [35].

### Preparation of whole cell lysates, cytosol, nuclear lysates, nuclear matrix lysates, and chromatin-binding protein lysates

Cells were transfected and harvested 72 hr post-transfection in a cell lysis buffer (50 mM Tris·HCl, pH 8.0, 0.5% Nonidet P-40, 2 mM EDTA, 137 mM NaCl, 10% glycerol, 0.5% sodium deoxycholate, 0.2% sodium azide, 0.004% sodium fluoride, 1× protease inhibitor mixture, 1 mM sodium orthovanadate, pH 7.25) for whole cell lysate. After incubation on ice for 20 min, whole cell lysates were obtained by centrifugation at 15,000 *g* for 10 min. To prepare cytosol and nuclear fractions, cells were resuspended in hypotonic buffer A (0.05% NP-40, 15mM Tris pH 8.0, 15mM NaCl, 60mM KCl, 1mM EDTA pH 8.0, 0.5mM EGTA pH 8.0), suspended, and incubated on ice for 5 min and centrifugated at 400 *g* for 5 minutes. The supernatant was collected as cytosol, the pellet was saved as nuclei. The nuclei pellet was suspended in buffer B (10mM Tris pH 8.0, 15mM NaCl, 60mM KCl, 1.5 mM EDTA pH 8.0) and incubated on ice for 20 min and centrifugated at 500 *g* for 5 minutes. The soluble fraction from buffer B extraction was collected as nuclear matrix lysates. The insoluble pellet from buffer B extraction was further suspended in Nuclear Extraction Buffer (5mM HEPES, 1.5mM MgCl2, 300 mM NaCl, 0.2mM EDTA, 0.5mM DTT adjust pH to 7.9), incubated on ice for 20 min, and centrifugated at 14,000 *g* for 10 min. The supernatant from this extraction was collected as chromatin-binding protein lysates. For nuclear lysates, the nuclei pellet was suspended in Nuclei Extraction Buffer (10mM Tris, pH 8.0, 250 mM NaCl, 1 mM EDTA), incubated on ice for 20 min on ice, and centrifugated at 14,000 *g* for 10 min. The supernatant from this extraction was collected as nuclear lysates.

### Immunoprecipitation, pulldown and Western Blotting

For immunoprecipitation, whole cell lysates (500 mg) were precleaned with 20 μl protein agarose beads and then incubated with 20 ul protein agarose beads with 1 μg antibody by rotation at 4^o^C overnight. For GST/His pulldown, 2.5 μg recombinant GST or GST-MEPCE protein was incubated with 2.5 μg Tip110.His protein in a volume of 500 μl Binding Buffer (20 mM HEPES, pH 7.9, 150 mM NaCl, 0.5 mM EDTA, 10% glycerol, 0.1% Triton X-100, and 1 mM DTT) containing 30 μl glutathione agarose beads (GST pulldown) or 30 μl Ni-NTA agarose beads (His pulldown). The beads were recovered by centrifugation and washed four times with Washing Buffer (50mM Tris, pH 8.0, 0.5% Nonidet P-40, 2mM EDTA, 0.4 M NaCl, and 10% glycerol). The beads were suspended in 1X SDS-PAGE sample loading buffer and boiled for 3 min for SDS-PAGE and Western Blotting.

### Glycerol Gradient ultracentrifugation

A glycerol gradient (10-40%) was made by sequentially loading 1.67 ml of each concentration of glycerol (10, 15, 20, 25, 30 and 40%, v/v) in PBS into a ultracentrifugation tube (Beckman). The tubes were placed at 4 ^o^C for 4 hr to allow gradient formation. Lysates as indicated were layered on the top of the glycerol gradients and spun in 186,000 *g* for 20 hr. Fractions (about 733 ul each for 14 fractions, about 1ml each for 10 fractions) were collected, proteins in each fraction were recovered by precipitation with 100% trichloroacetic acid, followed by twice with cold acetone, and then subjected to Western blotting against anti-CDK9 and HEXIM1 antibodies.

### RNA isolation and qPCR

Total RNA was isolated from transfected cells using TRIzol (Invitrogen). cDNA was synthesized using a Script kit (Biorad). qPCR was performed using qPCR SYBR Mix (Bio-Rad) and gene-specific primers. The q-PCR primers and their sequences are as follows: for β-actin: 5′-AAA CTG GAA CGG TGA AGG TG-3′ and 5′-AGA GAA GTG GGG TGG CTT TT-3′; for Tip110: 5′-GGC TAG GAT TGA GGC TCG ACT G-3′ and 5′-GGG TGT CAC CAT GAG CTC TTT CC-3′; for HEXIM1: 5’-AGC TGA CCT GGG AAG AGA A-3’ and 5’-GAG GAA CTG CGT GGT GTT ATA G-3’. β-actin was used as the loading control for qRT-PCR.

### Glutathione measurements

Total glutathione (GSH) and oxidized glutathione (GSSG) were measured using a Promega kit. Briefly, 293T cells were plated on a 96-well plate at a density of 2000 cells/well, cultured for 24 hr, and then transfected with 0.2 μg plasmid. Cells were lysed 72 hr post transfection in either Total Glutathione Lysis Reagent, which contains Luciferin-NT for the total GSH, or Oxidized Glutathione Lysis Reagent (Promega), which contains N-Ethylmaleimide and Luciferin-NT for GSSG. Luciferin Generation Reagent, which contains Glutathione-S-Transferase, was added to each well to be mixed and incubated for 30 min, followed by adding Luciferin Detection Reagent to each well to mix and incubated for 15 min. Luminescence was measured using a luminometer (Promega). Standards of different concentrations were included to obtain a standard curve and used to determine the concentrations in the samples.

### Fluorescence resonance energy transfer (FRET) microscopy

HeLa were plated and cultured in a 96-well glass bottom plate, transfected with plasmids as indicated using Lipofectamine 3000, and sensitized emission FRET measurements made 48 hr post transfection. The Cerulean and Venus constructs were excited sequentially and images captured using a PlanApo 60x, 1.42 NA oil immersion objective on an inverted microscope (IX71, Olympus America Inc.) and recorded on a CCD-based widefield fluorescence imaging system running the SimplePCI software (Hamamatsu). The Cerulean donor was excited at 430/24 nm and emission collected at 470/24 nM (donor channel) and 535/30 (FRET channel), and the Venus acceptor excited at 500/20 nm and its fluorescence emission collected at 535/30 (acceptor channel). The PixFRET plugin [36] for ImageJ was used to calculate donor and acceptor bleed-through (*BT_D_* and *BT_A_*, respectively) into the FRET channel and as well as normalized FRET (NFRET) values according to the formula [37]:

$$\mathrm{NFRET}=\frac{I_{\mathrm{FRET}}-I_{D}{\mathrm{BT}}_{D}-I_{A}{\mathrm{BT}}_{A}}{vI_{D}I_{A}}$$

Where *I_FRET_*, *I_D_*, and *I_A_* are the mean fluorescence intensities from a region of interest within the cell in the FRET, donor, and acceptor channel, respectively.

### Statistical analysis

The data are means +/- SD and representative of multiple independent experiments. All experiment data were analyzed by two-tailed student’s *t*-test. A *P* value <0.05 was considered to be statistically significant, and *P* value <0.005 was considered to be highly statistically significant.

## RESULTS

### Tip110 directly bound MEPCE

**Figure 1.**
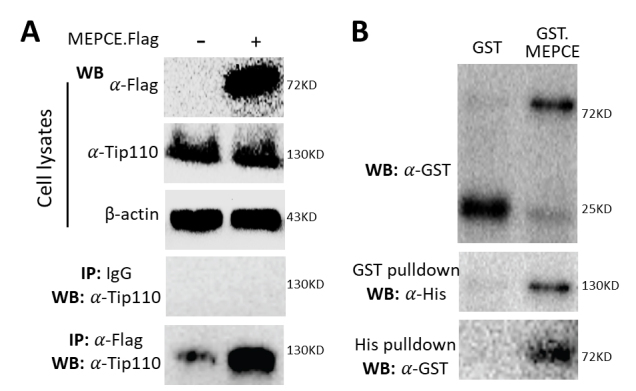
Tip110 binding to MEPCE. (A) 293T were transfected with pMEPCE.Flag and harvested for cell lysates, followed by Western blotting (WB) against anti-Flag, or anti-Tip110 antibody, or followed by immunoprecipitation (IP) against anti-Flag antibody or an isotype IgG, followed by Western blotting against anti-Tip110 antibody. pcDNA3 was included as a control for pMEPCE, while WB against anti-β-actin antibody was performed as an equal loading control. (B) Recombinant GST-MEPCE or GST was incubated with recombinant Tip110 His and analyzed by WB against anti-GST antibody, or pulled down with glutathione beads, followed by WB against anti-His antibody, or pulled down with Ni beads, followed by WB against anti-GST antibody.

Proteomics analysis leads to detection of Tip110 in the 7SK snRNP complex [38-40]. Our previous study demonstrates that there is no direct interaction between Tip110, CDK9 and cyclin T [34]. Thus, we determined if Tip110 would bind to other major components of the 7SK snRNP complex including LARP7, MEPCE, HEXIM1, and 7SK RNA. 293T were transfected with pMEPCE plasmid. Flag-tagged MEPCE and endogenous Tip110 expression were detected by Western blotting (Fig. 1A). Immunoprecipitation with anti-Flag antibody, followed by Western blotting against anti-Tip110 led to detection of Tip110 considerably more in MEPCE.Flag-expressing cells than the control. To further determine if there is a direct binding between Tip110 and MEPCE, recombinant Tip110 His was mixed with GST-MEPCE fusion protein or GST and subjected to GST pulldown, followed by WB against anti-His antibody. Tip110 was detected in the GST-MEPCE mixture, not in the GST mixture (Fig. 1B). Conversely, His pulldown and WB against anti-GST showed Tip110 in the GST-MEPEC mixture, not in the GST mixture. These results demonstrated that Tip110 bound to MEPCE in a direct manner. However, no direction binding between Tip110 and LARP7, HEXIM1 and 7SK RNA was detected (data not shown).

### Tip110 expression was associated with HEXIM1 and P-TEFb release from the 7SK snRNP complex

**Figure 2.**
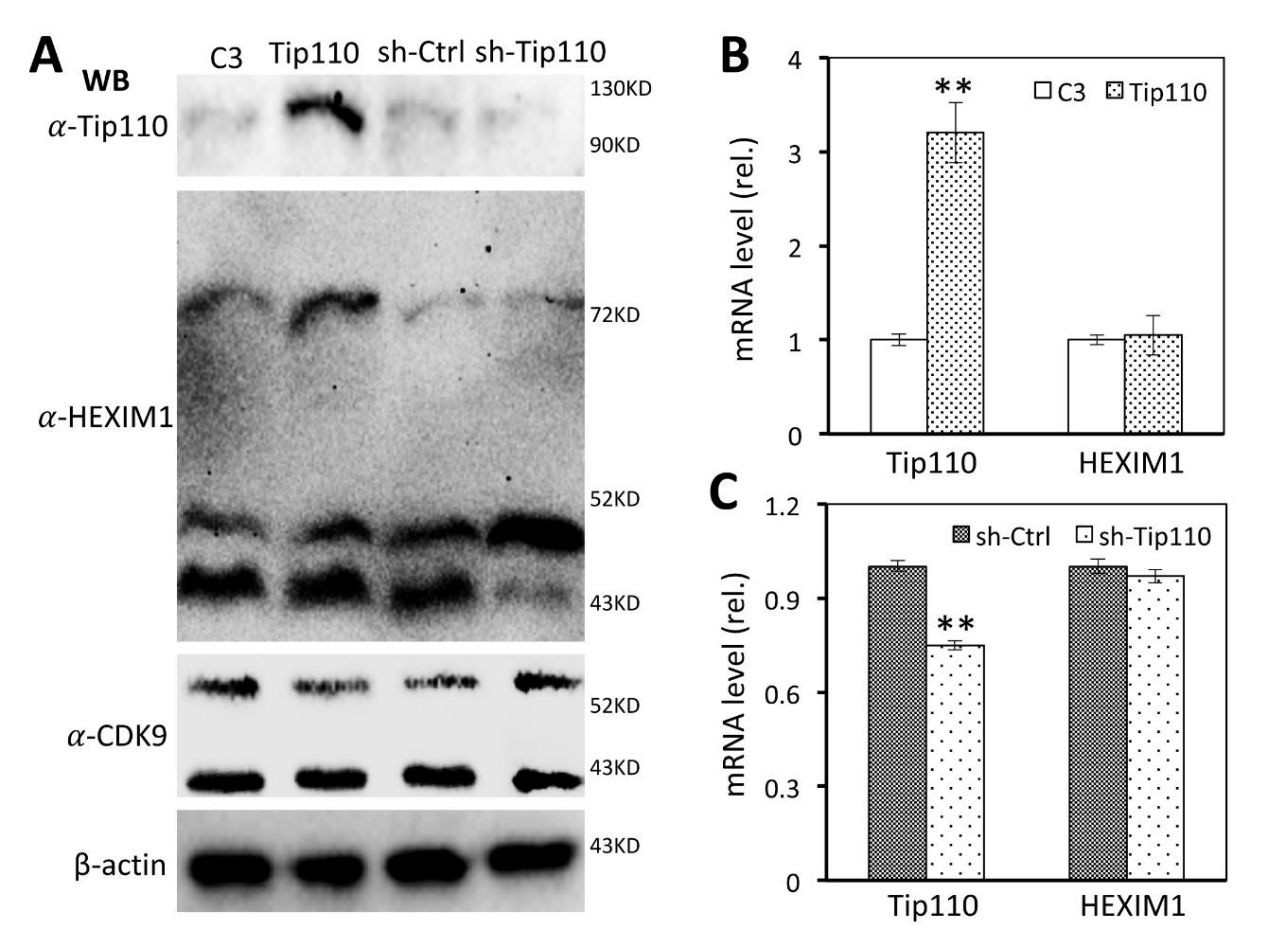
Tip110 expression had no effects on HEXIM and CDK9 expression. A. 293T were transfected with pTip110 or psh.Tip110 and harvested for cell lysates, followed by WB against anti-Tip110, anti-HEXIM, or anti-CDK9 antibody. pcDNA3 (C3) and pGIPZ (sh-Ctrl) were used as controls for pTip110 and psh.Tip110, respectively. WB against anti-β-actin antibody was performed as an equal loading control. B & C. Tip110 and HEXIM mRNA levels were determined by qRT-PCR in Tip110- or C3-transfected cells (B), or in sh-Tip110- or sh-Ctrl-transfected cells (C), normalized using β-actin, and calculated using C3 and sh-Ctrl as respective references.

**Figure 3.**
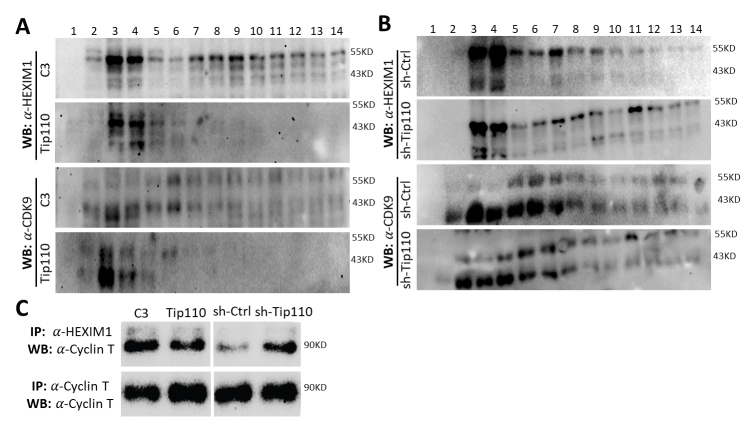
Tip110 expression altered association of HEXIM, CDK9 and Cyclin T. (A and B) 293T were transfected with pTip110 (A) or psh.Tip110 (B) and harvested for nuclear lysates. C3 and sh-Ctrl were used as controls for pTip110 and psh.Tip110, respectively. Nuclear lysates were loaded on the 10-40% glycerol gradient, separated by ultracentrifugation, and fractionated into 14 fractions. Each fraction was analyzed by WB against anti-HEXIM antibody, or anti-CDK9 antibody. C. Nuclear lysates obtained above were analyzed by IP against anti-HEXIM, or Cyclin T antibody, followed by WB against Cyclin T antibody.

To determine if Tip110 direct interaction with MEPCE that binds and stabilizes 7SK RNA would affect the 7SK snRNP complex formation, we modulated Tip110 expression by transfection of 293T with Tip110 plasmid for Tip110 over-expression or sh-Tip110 plasmid for shRNA-mediated Tip110 kockdown and determined HEXIM1 and CDK9 distribution among the fractions following by the glycerol gradient ultracentrifugation of nuclear lysates. pcDNA3 (C3) and psh-Ctrl transfections were included as the respective controls. First, whole cell lysates or total RNA were prepared from the transfected cells to determine whether Tip110 expresssion or knockdown would alter expression of the components of the 7SK snRNP complex. Western blotting confirmed that Tip110 over-expression or knockdown was detected and showed that there were no apparent changes in the expression levels of HEXIM1 and CDK9 proteins resulting from Tip110 over-expression or knockdown when compared to its respective control C3 or sh-Ctrl (Fig. 2A). Because of multiple bands of HEXIM1 detected on the anti-HEXIM1 antibody Western blot, qRT-PCR for Tip110 and HEXIM1 mRNA was further performed to confirm that the HEXIM1 mRNA expression level was not altered in response to Tip110 over-expression (Fig. 2B) or knockdown (Fig. 2C). Then, we prepared the nuclear matrix lysates, loaded them onto the 10-40% glycerol gradient, centrifuged, fractionated (14 fractions), and analyzed HEXIM1 and CDK9 in each fraction by Western blotting. Compared to the C3 control in which HEXIM1 was detected in two clusters, one around fraction #3, the other around fraction #9, Tip110 over-expression resulted in detection of HEXIM1 only in one cluster around fraction #3 (Fig. 3A). CDK9 exhibited similar distribution patterns to that of HEXIM1. To the contrary to Tip110 over-expression, sh-Tip110 knockdown led to detection of HEXIM1 in three clusters: one around fraction #3, one around fraction #7 and one around fraction #11, whereas the sh-Ctrl only had HEXIM1 in two clusters, one around fraction #3 and the other around fraction #7 (Fig. 3B). Again, CDK9 showed the similar distribution patterns as that of HEXIM1 in response to Tip110 knockdown. The concurrent shift of HEXIM1 and CDK9 from higher fractions to lower fractions by Tip110 over-expression and the concurrent shift of HEXIM1 and CDK9 from the lower fractions to higher fractions provided the first evidence that Tip110 is likely involved in regulation of the dynamic equilibrium of HEXIM1 and p-TEFb between the free form (smaller complex in the lower fractions) and the 7SK snRNP-bound form (larger complex in the higher fractions).

Both HEXIM1 and P-TEFb exist in two forms: free or in the 7SK snRNP complex. When they are free, they do not bind to each other, whereas they are in the 7SK snRNP complex, they bind to each other [1, 2, 39-42]. Therefore, a level of HEXIM1 that binds to CDK9 or cyclin T could be used as an indicator of the level of free (active) pTEFb. Thus, we further performed immunoprecipitation of the nuclear matrix lysates against HEXIM1, followed by Western blotting. Tip110 over-expression led to less HEXIM1-bound cyclin T compared to the C3 control, while Tip110 knockdown had more HEXIM1-bound cyclin T compared to the sh-Ctrol control (Fig. 3C). Immunoprecipitation of the same lysates against cyclin T, followed by Western blotting showed increased cyclin T in cells with Tip110 over-expression but no changes in cells with Tip110 knockdown. These results showed that Tip110 expression led to increased free (HEXIM1-unbound) cyclin T, while Tip110 knockdown showed less free (HEXIM1-unbound) cyclin T and further support the positive association between Tip110 expression level and the levels of free forms of HEXIM1 and pTEFb.

### Tip110 expression changed HEXIM1 dimerization and its localization to the chromatin

**Figure 4.**
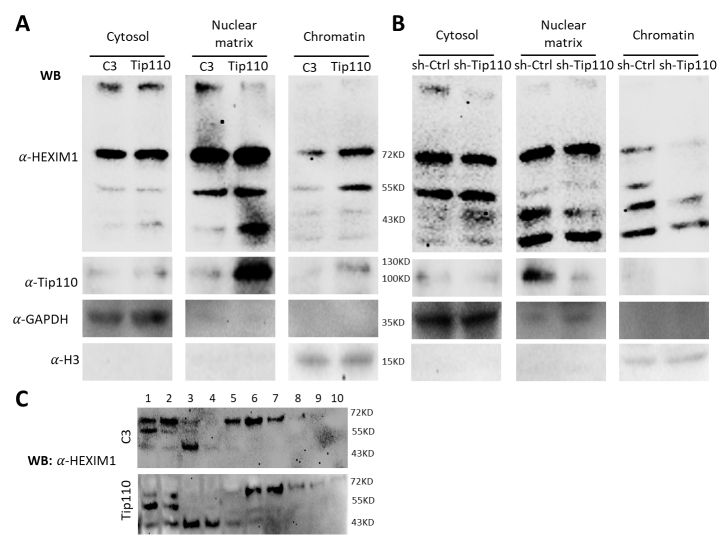
Tip110 expression altered HEXIM subnuclear localization. (A and B) 293T were transfected with pTip110 (A) or psh.Tip110 (B) and harvested for cytoplasmic lysates (Cytosol) and nuclear lysates. The latter was further separated into nuclear matrix lysates (Nuclear matrix) and chromatin-binding protein lysates (Chromatin). C3 and sh-Ctrl were used as controls for pTip110 and psh.Tip110, respectively. All lysates were analyzed by WB against anti-HEXIM, anti-Tip110, anti-GAPDH, or anti-Histone 3 (H3) antibody. C. Nuclear lysates from C3- or Tip110-transfected cells above were loaded on the 10-40% glycerol gradient, separated by ultracentrifugation, and fractionated into 10 fractions. Each fraction was analyzed by WB against anti-HEXIM antibody.

Our previous findings showed HEXIM1 as monomer, dimers and two unidentified species on the Western blot (Fig. 2A). Several studies have shown that the 7SK snRNP complex is tethered to the chromatin and serves as a readily available source of P-TEFb to activate the target gene promoter [1, 2, 41-44]. Thus, we further separated the cell lysates into three fractions: cytosol, nuclear matrix lysates and chromatin-binding protein lysates from Tip110- or sh-Tip110 transfected cells and analyzed HEXIM1 monomers/dimers and its localization by Western blotting. GAPDH and Histone 3 (H3) were included as the marker for cytosol and chromatin-binding protein lysates, respectively. Compared to the C3 control, Tip110 over-expression led to more HEXIM1 dimers in the chromatin-binding protein lysates (Fig. 4A). In contrast, Tip110 knockdown showed fewer HEXIM1 dimers in the chromatin-binding protein lysates compared to the sh-Ctrl (Fig. 4B). In addition, there were more HEXIM1 monomers in nuclear matrix lysates of Tip110-transfected cells and fewer HEXIM1 monomers in the chromatin-binding protein lysates of sh-Tip110-transfected cells. These results showed that Tip110 expression not only changed HEXIM1 monomer/dimer formation but also the localization of the HEXIM1-bound 7SK snRNP complex to the chromatin. To ascertain the effects of Tip110 expression on HEXIM1 monomer/dimer formation, we prepared nuclear lysates and analyzed the HEXIM1 distribution in the fractions (10 fractions) following a 10-30% glycerol gradient ultracentrifugation. DTT was used in the SDS-PAGE sample-loading buffer. Compared to the C3 control, Tip110 over-expression led to more monomer HEXIM1 in fractions 1-5 (Fig. 4C). These results provide additional evidence to support the notion that Tip110 expression regulates HEXIM1 oligomerization.

### Tip110 expression regulated the glutathione metabolic pathway and HEXIM1 dimerization/oligomerization

**Figure 5.**
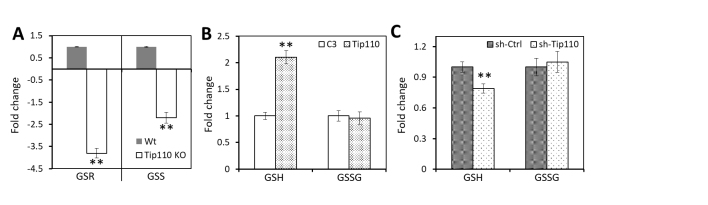
Effects of Tip110 on glutathione metabolism. (A) Relative expression levels of glutathione reductase (GSR) and glutathione synthetase (GSS) in Tip110-knocked out mouse embryonic stem cells. B & C. 293T were transfected with pTip110 (B) or psh.Tip110 (C), cultured for 48 hr, plated out in a 96-well plate, lysed, and assayed for total glutathione (GSH) and oxidized glutathione (GSSH). C3 and sh-Ctrl were used as controls for pTip110 and psh.Tip110, respectively and used as the references to calculate the fold change.

There are multiple cysteine residues within the primary amino acid sequence of HEXIM1, although the coiled-coil leucine zipper of HEXIM1 is involved in its dimerization [43, 44]. Thus, intramolecular and intermolecular disulfide bond formation between any two cysteine residues of HEXIM1 could also contribute to HEXIM1 dimerization and oligomerization. In separate transcriptomics studies, we have shown that Tip110 knockdown and knockout negatively regulated the glutathione metabolic pathway [45, 46]. Both mRNA of glutathione reductase (GSR) and glutathione synthetase (GSS), two major enzymes in the glutathione metabolic pathway were found to be 4 times and 2 times lower, respectively in Tip110 knockout embryonic stem cells than those in the Wt counterparts (Fig. 5A). To determine the direct effects of Tip110 expression on the glutathione metabolic pathway, we transfected 293T with Tip110 or sh-Tip110 plasmids and measured the total glutathione level (reduced form, GSH) and the oxidized glutathione level (oxidized form, GSSG) in these cells. Tip110 over-expression increased GSH by about 2.1-fold over the C3 control (Fig. 5B), while sh-Tip110 knockdown decreased GSH by 18% over the sh-Ctrl (Fig. 5C). However, there were no significant changes in GSSG in both Tip110- and sh-Tip110-transfected cells.

**Figure 6.**
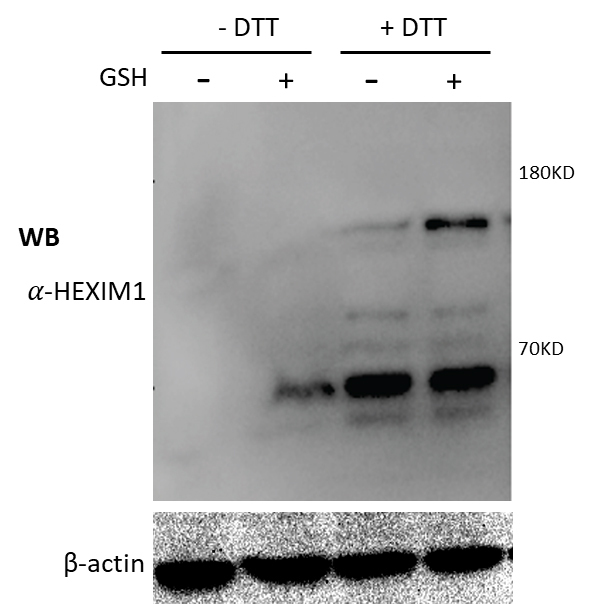
Direct effects of glutathione on HEIXM dimerization/oligomerization. 293T were cultured in the absence or presence of 100 uM GSH for 2 hr and then harvested for nuclear lysates. The nuclear lysates were treated with 4X SDS-PAGE sample buffer containing no DTT or 400 μM DTT and analyzed by WB against anti-HEXIM antibody. WB against β-actin antibody was included as an equal loading control.

We next determined if glutathione would affect HEXIM1 oligomerization *in vivo* by treating the cells with GSH and analyzed HEXIM1 oligomerization using Western blotting of the nuclear lysates. When no DTT was added to the SDS-PAGE sample-loading buffer, there was barely any monomer/dimer HEXIM1 detection on the cells treated without GSH and only monomer HEXIM1 detection in the cells treated with GSH (Fig. 6). However, when DDT was included in the SDS-PAGE sample-loading buffer, there were comparable levels of monomer HEXIM1 detection in the cells both treated with or without GSH. In addition, there was also increased dimer HEXIM1 detection in the cells treated with GSH. Similar results were obtained using purified recombinant HEXIM1 protein treated with or without GSH (data not shown). These results showed that Tip110 expression regulated HEXIM1 dimerization/oligomerization and that changes of glutathione-mediated intracellular redox level might be involved in this regulation. These results also indicate that lack of HEXIM1 detection on the Western blot in the absence of DTT and GSH is likely because HEXIM1 is heterogeneously oligomerized to become too diffused to be detected.

### Tip110 expression-induced HEXIM1 dimerization/ oligomerization by FRET

**Figure 7.**
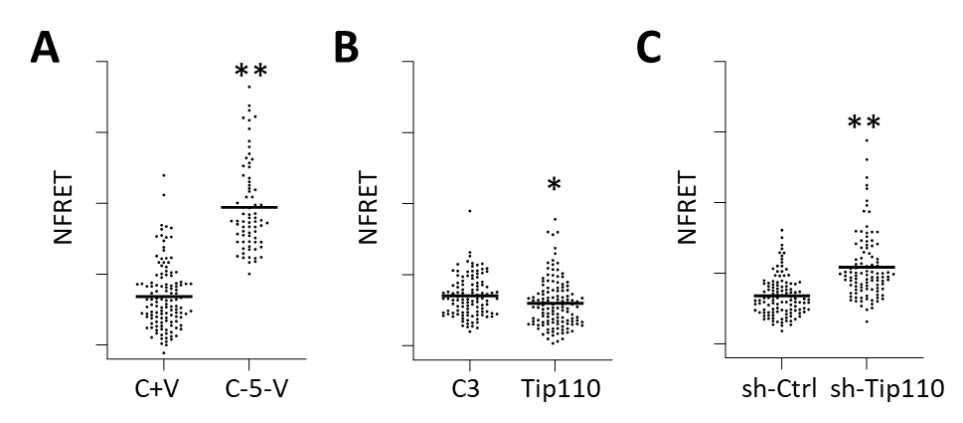
Effects of Tip110 expression on HEXIM dimerization/oligomerization by FRET. (A) pCerulean and pVenus (C+V), or pCerulean-5-Venus (C-5-V) were transfected into Hela to serve as negative and positive controls for FRET analyses. (B and C) Hela were transfected with C-HEXIM and V-HEXIM, C3, or Tip110 (B), or C-HEXIM and V-HEXIM, sh-Ctrl, or sh-Tip110 (C). All transfected cells were subjected to fluorescence microscopic analysis 48 hr post transfection.

To confirm that Tip110 expression is associated with HEXIM1 dimerization/oligomerization *in vivo*, we transfected Hela with Cerulean-HEXIM1 and Venus-HEXIM1 in combination with Tip110, sh-Tip110, or its respective control and performed the FRET microscopic analysis. Cerulean plus Venus plasmid (C+V) and Cerulean-5-Venus (C-5-V) plasmid were also transfected and used as negative and positive FRET controls, respectively (Fig. 7A). Compared to the C3 control, Tip110 over-expression led to lower FRET (Fig. 7B). In contrast, Tip110 knockdown gave rise to higher FRET over the sh-Ctrl (Fig. 7C). Consistent with previous findings, these results demonstrated that Tip110 over-expression led to more monomer HEXIM1, whereas Tip110 knockdown led to more dimer/oligomer HEXIM1.

## DISCUSSION

In this study, we reported a new mechanism regulating HEXIM1 dimerization/oligomerization by Tip110. There are several lines of evidence to support this new mechanism. Tip110 over-expression showed more HEXIM1 in the lower fractions (smaller complex) of the glycerol gradient, while Tip110 knockdown had more HEXIM1 in the higher fractions (larger complex) of the glycerol gradient. This positive association between Tip110 expression and HEXIM1 monomer/dimer/ oligomer formation was found in the glycerol gradient of nuclear matrix lysates, the glycerol gradient of nuclear lysates, as well as in the chromatin-binding protein lysates. HEXIM1 changes from dimer/oligomer to monomer in Tip110-expressing cells and from dimer to oligomer in Tip110-knoced down cells were further validated using FRET microscopy. In addition, there was concurrent shift of CDK9/cyclin T from the larger complex to smaller complex when Tip110 was over-expressed and concurrent shift of CDK9/cyclin T from the smaller complex to larger complex when Tip110 was knocked down. HEXIM1 dimerization or oligomerization is essential for inhibiting P-TEFb in that each HEXIM1 dimer binds to two P-TEFb and HEXIM1 oligomer binds to P-TEFb in the same ratio. HEXIM1 dimer or oligomer binds to one 7SK RNA, which is extremely important to hold P-TEFb in the 7SK snRNP complex and efficiently inactivate P-TEFb [1, 42-44]. It is conceivable that when HEXIM1 dimer or oligomer disassembles to become monomer, HEXIM1 is unable to incorporated into the 7SK snRNP complex. Meanwhile, P-TEFb is no longer bound to HEXIM1 and released from the 7SK RNP complex to be in active state. The free monomer of HEXIM1 is able to form dimer again and to bind to the 7SK RNA. However, it is not still clear whether CDK9/cyclin T release from or assemble onto the 7SK snRNP complex is a result of HEXIM1 shift from dimer/oligomer to monomer.

HEXIM1 has been shown to form dimers or oligomers through the coiled-coil leucine zipper in its C-terminal domain [43, 48]. Leucine zipper has the great versatility as they can facilitate formation of homo- or hetero-dimer, trimer, or tetramer [47, 49-52]. This motif forms a continuous α-helix that can dimerize through formation of a coiled-coil structure involving paired contacts between the hydrophobic leucine zipper domain. In this study, we demonstrated that Tip110 expression-mediated changes in glutathione metabolic pathway, or specifically the intracellular redox level could also contribute to HEXIM1 oligomerization. Tip110 knockdown and knockout transcriptomics analyses independently identified changes of glutathione metabolism in Tip110 knockdown and knockout cells. Two major enzymes GSR and GSS showed significant down-modulation in Tip110 knockout cells. There was positive association between Tip110 expression and intracellular GSH production. Importantly, GSH treatment led to formation of HEXIM1 monomers. GSH is the most abundant and versatile low molecular weight thiol in the cell. When oxidized to GSSG, it is either extruded from the cell without metabolism (as GSSG) or reduced back to GSH via glutathione reductase by reducing equivalents derived from the pentose shunt [53-55]. A large number of proteins have been reported to be modified by GSH. This modification could change the catalytic or structural properties of proteins, affect protein oligomerization, or alter ligand binding, or by promoting inter-protein thiol disulfide exchange, stimulate the formation of cystine-linked protein oligomers [53-56]. Redox-dependent oligomerization through a leucine zipper motif also occurs [55].

Tip110 binds to a number of proteins and regulates gene expression, they include Tat, RNAPII, androgen receptor, YB-1, USP4, USP15 [57-60]. We showed in this study that Tip110 directly bound to MEPCE, which functions to methylate and stabilize the 7SK RNA. Tip110 is detected in the nuclear speckles [34] Interestingly, 7SK snRNA has also been localized in the same nuclear speckle structure [18]. Thus, it is reasonable to conclude that Tip110 is present in the 7SK snRNP complex, controls HEXIM1 and P-TEFb release from the 7SK snRNP complex and activates P-TEFb target gene expression through regulation of the intracellular redox level. These findings also uncover one additional unique mechanism for the roles of Tip110 in HIV gene transcription and replication.
