# Erratum to “Tip110 expression facilitates the release of HEXIM1 and pTEFb from the 7SK ribonucleoprotein complex involving regulation of the intracellular redox level”

**DOI:** 10.14336/AD.2023.0504

**Published:** 2023-06-01

**Authors:** Ying Liu, Lu Li, Khalid Timani, Carl White, Johnny J He

We have noticed inadvertent errors in our article published in the December 2021 issue of Aging Dis (2021, 12(8) 2113-2124). The images of Figure 2A (CDK9) and 4B (Tip110) have been presented incorrectly. We have attached the corrections below. The errors do not change the scientific conclusions of the article. The authors would like to apologize for the errors and any inconvenience caused.

**Figure 2A.**
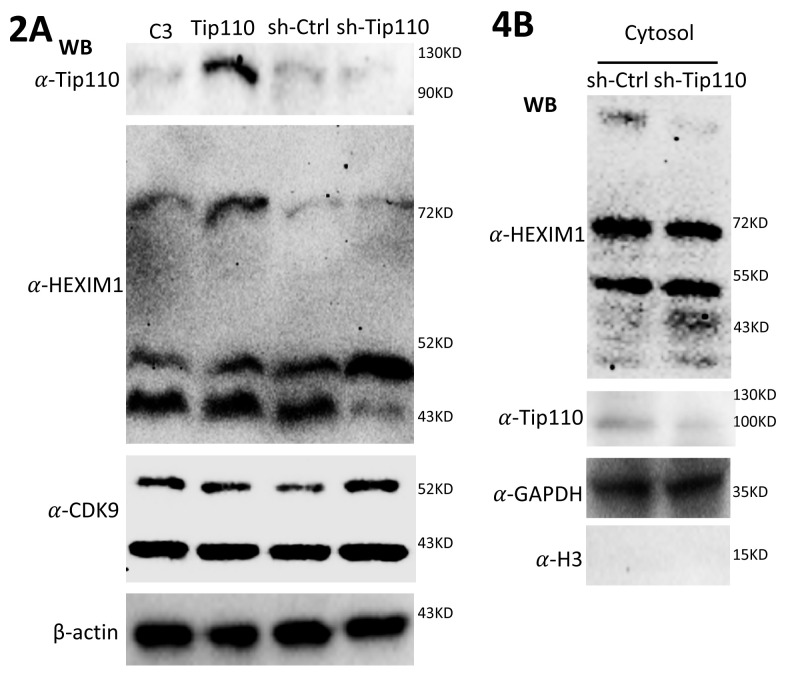
293T were transfected with pTip110 or psh.Tip110 and harvested for cell lysates, followed by WB against anti-Tip110, anti-HEXIM, or anti-CDK9 antibody. pcDNA3 (C3) and pGIPZ (sh-Ctrl) were used as controls for pTip110 and psh.Tip110, respectively. WB against anti-β-actin antibody was performed as an equal loading control.

**Figure 4B.**
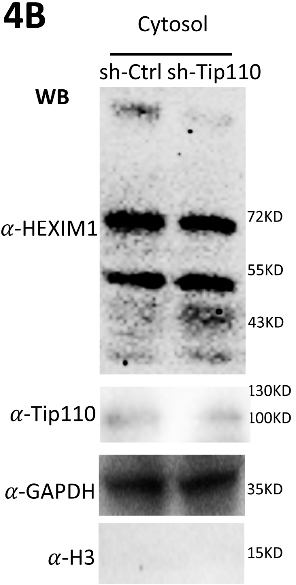
293T were transfected with psh.Tip110 and harvested for cytoplasmic lysates (Cytosol). sh-Ctrl was used as controls for psh.Tip110. Cytosol lysates were analyzed by WB against anti-HEXIM, anti-Tip110, anti-GAPDH, or anti-Histone 3 (H3) antibody.

